# Like milk on the stove: Healthcare professionals navigating uncertainty when caring for families with 22q11DS

**DOI:** 10.1371/journal.pone.0313845

**Published:** 2025-01-09

**Authors:** Sophie Ayoub, Eva De Clercq, Cheryl Cytrynbaum, Luzius A. Steiner, Bernice S. Elger

**Affiliations:** 1 Institute for Biomedical Ethics, University of Basel, Basel, Switzerland; 2 Division of Clinical & Metabolic Genetics and the Department of Genetic Counselling, The Hospital for Sick Children, Toronto, Canada; 3 Department of Molecular Genetics, University of Toronto, Toronto, Canada; 4 Department of Anesthesiology, University Hospital Basel, Basel, Switzerland; 5 Department of Clinical Research, University of Basel, Basel, Switzerland; 6 Center for Legal Medicine, Faculty of Medicine, University of Geneva, Geneva, Switzerland; Flinders University, AUSTRALIA

## Abstract

**Introduction:**

22q11 deletion syndrome (22q11DS) results from a microdeletion on chromosome 22 and is the most common microdeletion disorder in humans, affecting 1 in 2148 live births. Clinical manifestations vary widely among individuals and across different life stages. Effective management requires the involvement of a specialized multidisciplinary team. This study aims to explore the experiences of healthcare professionals in caring for the families of children with 22q11DS, focusing on their challenges, rewards, and coping strategies.

**Methods:**

Data for this interview study were collected as part of a broader mixed methods research project aimed at enhancing the psychosocial well-being of children aged 3–15 years with 22q11DS and their families. The qualitative aspect of this study focused on capturing the experiences of healthcare professionals involved in their care, recruited purposively through collaborators and snowball sampling methods. Reflexive thematic analysis of semi-structured interviews was performed after verbatim transcription.

**Results:**

Twenty healthcare providers from different specialties were interviewed. The majority had a working experience of more than 10 years and were part of a 22q11DS clinic. After data analysis, four themes (and many sub-themes) were identified that were all related to the topic of uncertainty: acknowledging uncertainty, sharing uncertainty, acting on uncertainty and coping with uncertainty. Many experts showed a sense of humbleness when caring for the families and most of the participants emphasized the role of peer support and multidisciplinary teams.

**Conclusion:**

Our study reveals how healthcare professionals manage the uncertainty associated with 22q11DS, highlighting the importance of peer support and multidisciplinary team collaboration. Providers recognize the limits of their medical expertise and value the perspectives of families living with the condition. Their coping strategies play a critical role in handling uncertainty and suggest a need for further emphasis in the literature on the experiences of healthcare professionals dealing with rare diseases.

## Introduction

22q11 deletion syndrome (22q11DS), also known as DiGeorge syndrome, results from a microdeletion on the long arm of chromosome 22 [1]. It is a rare disease and the most common microdeletion disorder in humans, occurring in approximately 1 in 2148 live births [2]. The syndrome is characterized by a broad range of clinical manifestations that vary significantly among individuals and across different stages of life. Common manifestations include congenital cardiac defects and swallowing difficulties in infancy, immunological abnormalities, speech and language delays, cognitive disabilities in childhood, and neuropsychiatric conditions in later years [3–5].

In light of this diverse spectrum of symptoms, managing 22q11DS necessitates the involvement of a specialized multidisciplinary team (MDT) that can address both medical needs and long-term developmental concerns [3–5]. MDTs typically include geneticists, cardiologists, immunologists, speech and language therapists, neurologists, psychiatrists, and social workers [6].

To enable parents to better understand the condition and the importance of regular monitoring and early intervention, healthcare professionals (HCPs) must provide comprehensive education about the syndrome. This education empowers families to make informed decisions and prepares them for the potential challenges ahead [7].

In addition, professionals should offer psychological support to help families cope with the emotional and social challenges associated with the syndrome [8, 9].

Previous work by Garrino et al. described the challenges experienced by HCPs in trying to navigate their own emotions, including frustration and discouragement, when they witness the suffering experienced by their patients, giving rise to feelings of impotence. This results in considerable emotional burden and heavy workload for healthcare staff who have to already fulfill many roles in the healthcare system as administrators, researchers and clinicians. This leads to excessive engagement that can occasionally make it challenging to remain clear-headed in treatment decisions [10] and to preserve the quality of care.

In order to develop sustainable family-centered care, a healthcare approach that is attuned to, meets and respects not only the needs and values of the affected individual (patient-centered) but of their whole family who supports them [11], it is important to take into account the viewpoint of both families and providers to effectively meet their needs, values and, preferences.

Existing research on rare diseases, particularly on 22q11DS, often centers on medical diagnosis and management by HCPs rather than on their care-giving experiences [7, 12, 13].

In this study, we aim to explore HCPs’ experiences in caring for families where there is a child with 22q11DS. Specifically, we seek to explore professionals’ perceptions of the challenges and rewarding experiences they encounter, and the strategies they use to cope. To our knowledge, this study represents one of the first qualitative inquiries into HCPs’ care experiences in the context of 22q11DS care and rare diseases in general, and provides valuable insights into how best to improve care for this patient population.

## Methods

The data for this interview study were collected as part of a broader mixed methods research project that aimed to improve the psychosocial well-being of children aged 3–15 years with 22q11DS and their families. The qualitative part of the study aimed to capture the caregiving experiences of parents and HCPs.

Since the participating parents and HCPs were not responsible for taking care of the same child, no correlation was possible.

### 1. Sample

The sample for this part of the interview study consisted of 20 HCPs in Europe and Canada, who were involved in the care of children (3–15 years) with 22q11DS, with at least 1 year of work experience in the field. They were recruited purposefully through collaborators on the project (N = 2), and snowball sampling (N = 10). To reach 20 HCPs, the first author SA sent an email to 21 professionals who are board members of the main 22q11DS organizations (22q11europe, 22q and 22q society) and had valid contact details, summarizing the research project and inviting them to an interview. Of the ones who answered (N = 11), 6 were eligible and were interviewed, and they provided contact details for 2 other interviewees.

### 2. Ethics approval

The study was approved by the Ethics Committee of the University of (—blinding) for research with human subjects. The study was conducted in compliance with the protocol, the current version of the Declaration of Helsinki, the ICH-GCP, and ISO EN 14155 (as far as applicable) as well as all national legal and regulatory requirements. The data were stored in accordance with the General Data Protection Regulation (GDPR) on a secure university server and were only accessible to the research team.

### 3. Data collection

The recruitment started on October 19, 2022, and interviews were conducted until April 21, 2023. HCPs were contacted via email by the interviewer SA (no prior relationship between both parties) and were sent an information sheet. Written informed consent was obtained from each participant. All interviews were done via the digital platform Zoom by the same researcher SA. SA is a female medical doctor working on her PhD project and trained to conduct qualitative research. Interviews were held in English and were between 40 and 77 minutes in length. First, the purpose of the study, minimal risks, and benefits were explained. Then, respect for privacy and confidentiality were discussed before starting with a semi-structured interview (with questions and prompts) following an interview guide that was pilot tested. Questions focused on HCPs’ caregiving experience, perceived needs of families, available support services, perceived impact of the condition on families, and coping experience of HCPs. The interviews also touched upon the HCPs’ perceptions of the future and an online intervention tool (newly developed that improves children’s well-being, enhances parental skills, and reduces parental distress).

### 4. Data analysis

All interviews were recorded with consent, transcribed, and then inductively coded using the qualitative analysis software MAXQDA 24 to support structured data analysis. All personal or situation related information possibly permitting identification was de-identified following verbatim transcription to maintain anonymity and respect for privacy and confidentiality. Coding started simultaneously with transcription. Two interviews were coded simultaneously by SA and EDC, which resulted in most codes being congruent. The rest of the interviews were coded solely by SA. Reflexive thematic analysis (RTA) by Braun and Clark [14] was used by the above-mentioned researchers to analyze the empirical data. Reflexive thematic analysis is a qualitative research method used to identify, analyze, and report patterns (themes) within data. This approach involves immersing oneself in the data, coding to identify key features, and refining these codes into themes through a process of constant reflection and iteration. Throughout, the researcher maintains a reflexive stance, acknowledging their influence on the analysis to ensure a nuanced and interpretive account of the data [14, 15].

*Researcher reflexitivity: As a researcher with a clinical background (not in RDs), she remained mindful of how her professional experiences might shape data analysis. To mitigate bias, she regularly reflected on her assumptions through researching and reading and especially through analytical discussions with the other members of the research team who had all different specialties, ranging from theology, philosophy to genetics and healthcare management. This allowed her to stay open to participants’ narratives and ensure the analysis reflected their experiences rather than her preconceptions.*

## Results

For this study, we interviewed twenty HCPs (16 females and 4 males), including 2 genetic counselors, 4 psychologists, 2 psychiatrists, 2 ear-nose-and-throat (ENT) surgeons, 3 pediatricians, 4 speech-language pathologists, 1 clinical immunologist, 1 nurse-coordinator and 1 occupational therapist. The majority had a working experience of more than 10 years and were part of a 22q11DS clinic. They were based in either Canada or Europe (see Table 1).

**Table 1:** Demographics of interviewed healthcare professionals.

HCP	Sex	Specialty	Years of experience with 22q11	Clinic	Number of children seen in consultation
HCP1	F^(1)^	Genetic counselor	25 years	22q clinic	6-10/month
HCP2	F	SLP^(3)^	40 years	22q clinic	4-5/month
HCP3	F	Psychologist	30 years	22q clinic	450/year
HCP4	F	Psychologist	5 years	22q clinic	20/year
HCP5	M^(2)^	ENT surgeon	25 years	University hospital	2-3/month
HCP6	F	SLP	24 years	22q clinic	3-4/month
HCP7	F	Nurse coordinator	25 years	22q clinic	NA
HCP8	F	Genetic counselor	18 years	22q clinic	6-10/month
HCP9	F	Child psychiatrist	4 years	General clinic	2/month
HCP10	F	ENT surgeon	12 years	University hospital	5-10/month
HCP11	M	Pediatrician	36 years	22q clinic	450 in total
HCP12	F	Pediatrician	30 years	22q clinic	6-10/month
HCP13	M	Clinical immunologist	25 years	General clinic	2-3/year
HCP14	F	Psychologist	12 years	22q clinic	5/month
HCP15	M	Psychiatrist	23 years	22q research clinic	20/year in total
HCP16	F	SLP	12 years	22q clinic	2/week
HCP17	F	Occupational therapist	10 years	general clinic	2-3/year
HCP18	F	Psychologist	17 years	22q clinic	6-7/month
HCP19	F	Pediatrician	6 years	22q clinic	2/week
HCP20	F	SLP	25 years	22q clinic	0-5/week

^(1)^ Female;

^(2)^ Male;

^(3)^ Speech-language pathologist;

**Fig 1:**
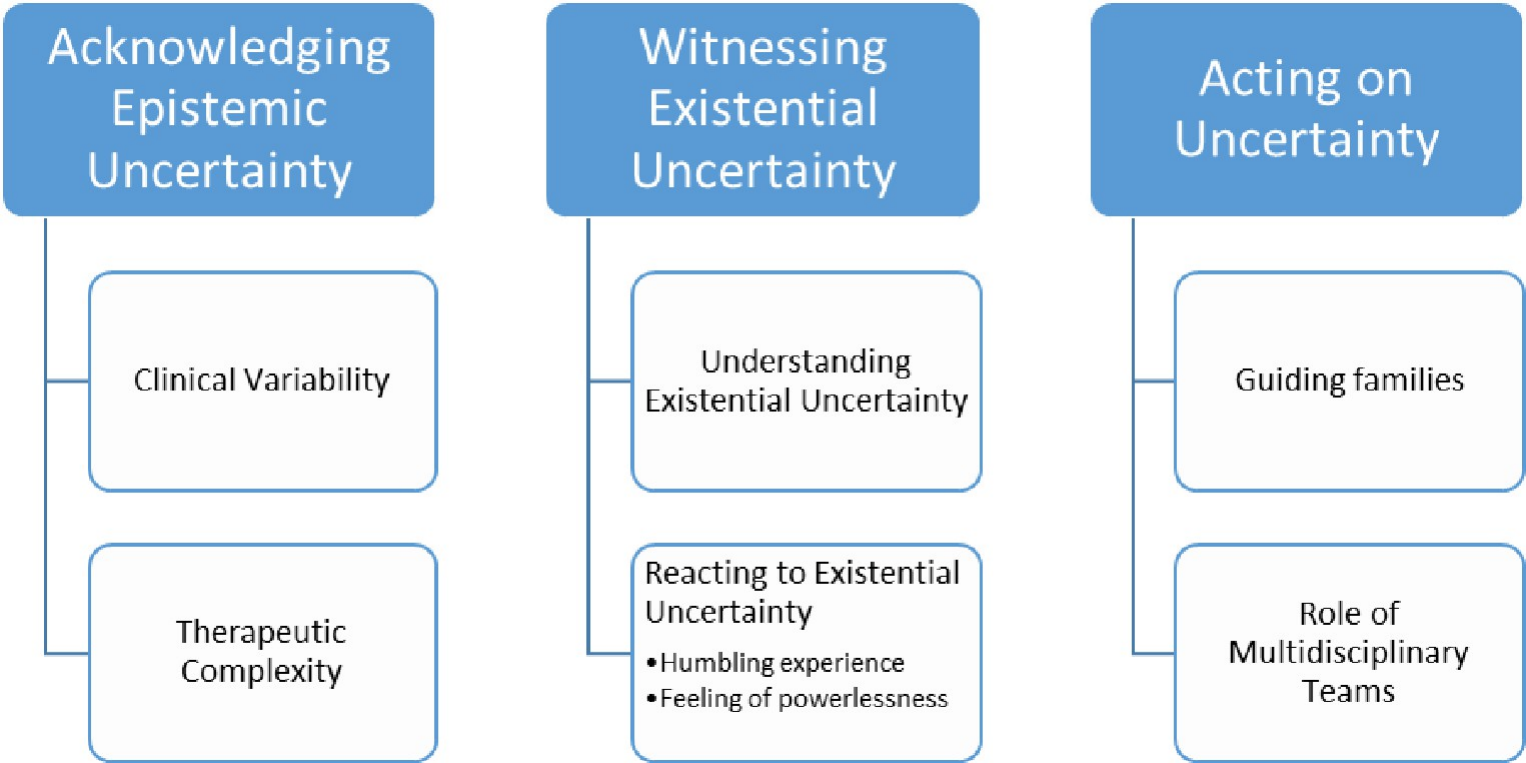
Themes and subthemes.

After data analysis, three themes were identified that were all related to the topic of uncertainty. This theme took on different meanings (see Fig 1): 1. Acknowledging epistemic uncertainty 2. Witnessing existential uncertainty 3. Acting on uncertainty. Representative and anonymized quotes were taken from each interview to illustrate the findings.

### 1. Acknowledging epistemic uncertainty

Epistemic uncertainty arose mainly due to the limitation of medical knowledge on 22q11DS (rather than due to HCP’s personal inadequacy). All HCPs acknowledged the presence of epistemic uncertainty in both the clinical manifestation and the treatment of 22q11DS. The variability of clinical manifestations between individuals, and within one individual’s lifespan and the fact that therapeutic options are not always effective for all patients, led our HCPs to recognize the medical complexity of the condition.

#### 1.1. Clinical variability

The majority of experts emphasized that the clinical presentation of the syndrome is broad and highly variable in patients, ranging from minimal symptoms to multiple medical complexities, and varying from life-threatening conditions to very mild presentation. They reported that a diagnosis may be established in infancy, for those with congenital cardiac lesions or feeding problems, while others may be diagnosed in childhood who present with speech delay and some may not be diagnosed until adulthood, as their symptoms were not recognized or investigated. In addition to physical features, experts highlighted that patients have variable degrees of cognitive disabilities as well as an increased risk to develop mental health disorders. They argued that this broad spectrum of symptoms makes the clinical presentation uncertain and complex to grasp.

*HCP1 (genetic counselor): So I think that one of the most important things to recognize about 22q11DS is that it is a broad spectrum (…) So there are tremendous variable medical presentations from children who need lifesaving heart surgery in the newborn period to individuals who may have minimal medical manifestations throughout their whole life (…) as well from a neurodevelopmental perspective and mental health (…) There are people who might have mild learning disabilities that have completed college degrees, and individuals who have a diagnosis of autism spectrum disorder and intellectual disability who are not able to complete a high school diploma.*

*HCP20 (speech-language pathologist)*: *It’s different from child to child how many problems they have*, *I mean some of them are really ill*, *and have a lot of immune deficiencies and cardiac problems and heart surgeries when they are small (…) then we have the other part of children who don’t have a diagnosis as young children but a little bit later and they often haven’t had so much severe problems*.

Given this wide array of unique health scenarios, experts highlighted the importance of individualized follow-up care. Healthcare providers expressed the hope of delivering targeted and comprehensive care plans, but did not always succeed, due to a lack of time and resources. Adjustments to treatment strategies were believed to be essential to address evolving health concerns and optimize overall well-being.

*HCP2 (speech-language pathologist): These individuals possibly have, you know, difficulties in multiple areas and many times deciding what the child needs can be contingent on what else they need (…) I’m not going to recommend speech surgery if the child needs cardiac surgery within the next two months, so communicating with disciplines, knowing that they need this, but they also have fluid in their ears and need tubes at the same time. So again we can really figure out the timing to make sure that it makes sense and making sure that we’ve looked up every area so that we’re looking at a whole child.*

*HCP4 (psychologist)*: *We see kids who probably have very severe intellectual disabilities all the way up to average intelligence (…) I’d say it’s challenging for us because we can’t do follow-ups*, *so we want to be able to follow up with these kids because we recommend they have a follow-up assessment*, *and we think it’s very important to follow-up their development over time*, *but we can’t really offer that*. *So I think it’s really frustrating to us that we can’t really see them several times over the course of their development to catch problems early*, *especially during adolescence*, *when you know*, *the possibility of psychosis becomes an issue for some kids*. *We’re not there for them to look at these early warning signs or be able to intervene quickly*.

In addition to clinical heterogeneity among patients, HCPs also referred to the changing clinical situation of each pediatric patient over time. They often described a fluctuating health scenario in which families seemed to have found some stability only to discover new clinical complexities, not long afterwards. They highlighted that families’ care needs change across the lifespan and that HCPs need to focus more on the somatic side of the condition when patients are young and more on the developmental and psychosocial aspects when caring for older pediatric patients. Many of them emphasized the difficulty of making accurate prognostic evaluations. Of particular concern was the uncertainty regarding the neuropsychiatric trajectory.

*HCP11 (pediatrician): Obviously, in infancy, perhaps particularly for feeding issues or developmental issues, we would see them probably 2, 3, 4 times a year together with our clinical specialists. Then, depending on whether the issue is speech and language, they would be seen by speech language therapists and surgeons between the age of 3 and 5 quite intensively, then usually by the age of 7 (…) we reach a level of speaking that we could be happy with, and during that period, the preschool period, they would also get the psychological assessment and to some extent have some input from psychologists and psychiatrists, if there are some behavioral issues, which of course are very frequent.*

*HCP16 (speech-language pathologist): The children that we follow up often have disorders impacting their physical health like immune-related disorders, immunity problems, or growth deficiencies, or feeding difficulties, and as well as their mental health. That’s Professor (name) who would steer them more. But sometimes parents report to us that their behavior is changing (…) and we look if we need to provide more diagnosis for that and to the children the correct care (…) We need to think how we can help them as a team in the best way possible*.

*HCP18 (psychologist): When we see parents of relatively young kids, often the families are very focused on the medical aspects like the surgeries the palatal abnormalities (…) Then when they start school after 2 or 3 years, the learning challenges come at the forefront and then eventually when they are becoming teenagers, it’s more often the behavioral aspects or the psychiatric aspects that come at the forefront. So it’s always a bit of a different topic and when they become adults, of course, there are a lot of questions around autonomy, work, sexuality, relationships. So definitely it’s not at all the same conversations when we are talking with parents who have a young kid and an older one*.

*HCP19 (pediatrician)*: *They (parents)’re usually looking for answers to what will happen later on and how should I deal with this and what kind of help could I get from the society*. *And for some of them who are diagnosed neonatally*, *they have a lot of physical issues in the beginning*, *so some of the parents are asking about the neuropsychiatric probabilities later on or the possibility for intellectual disabilities*, *but they also very soon settle with that we don’t know and that we have to wait and see*.

### 1.2. Therapeutic complexity

In terms of treatment, many participants were aware of the inability to offer families a guarantee of therapeutic success. This was particularly true for unintelligible speech, where they found it difficult to ensure a clear speech prognosis after surgery or therapy because of possible coordination and learning problems. Some experts highlighted that corrective surgery of speech is complicated due to the distinctive anatomy of individuals with 22q11DS.

*HCP5 (ENT surgeon): So when we do for example a palate repair that’s usually very rewarding, but it does not always work I would say that about 30% of the cases will need some subsequent surgery to help them with their speech and then after that, making sure that we do the right operation to help them with their speech is also a challenge (…) If I did get bleeding from an internal carotid artery in the back of the throat, it would likely be fatal. Now I’ve never had that, but it’s one of those theoretical things and I always talk to the families about it (…) And I never had to cancel a surgery, but I always warn them there is a very slight chance.*

*HCP10 (ENT surgeon)*: *(…) Then also of course the speech prognosis is even after an operation quite unpredictable because there are some coordination and learning issues*, *and it sometimes takes longer after an operation to see an improvement and that can be sometimes difficult because with some children it’s very variable in post-operative outcome*.

Some participants also considered the challenges surrounding the treatment of mental health problems, especially in children with multiple comorbidities like 22q11DS, to be multifactorial. First, pharmacological therapy was said to possibly interact with other health conditions, complicating treatment approaches. Additionally, one expert believed that the hesitation of some HCPs to offer pharmacological therapy to children, despite biological factors indicating its potential efficacy, could stem from other sources, including cultural perceptions.

*HCP9 (psychiatrist): Some of the kids are quite medically complex (…) We’ve had kids with quite severe cardiac and pulmonary hypertension, is it safe to put them on psychostimulants??? that kind of questions.*

*HCP18 (psychologist)*: *I think a very (city) issue (…) it is that when we deal with a child or adolescent*, *people are quite reluctant to treat with medications which I can understand*, *because if we can avoid medications of course we should*! *But I think in those specific conditions*, *there is really a strong biological background*, *then sometimes we need medications*. *So for example*, *for attention difficulties*, *in (city)*, *it’s incredibly difficult to get a prescription for psychostimulants*, *and if there are like major attentional difficulties*, *that can really have a strong impact on learning in school*, *it is still difficult to get a prescription and to have the child treated and that’s a very strong barrier I would say*.

### 2. Witnessing existential uncertainty

HCPs witnessed the existential uncertainty experienced by parents of children with 22q11DS with empathy and understanding. They understood the profound emotional impact of this uncertainty on parents and their families, recognizing the complex blend of fear, anxiety, and hope that accompanies it.

#### 2.1. Understanding existential uncertainty

Most participants said they found it challenging to communicate the diagnosis of 22q11DS to families, because they were aware that they were not only destroying parents’ dream of a healthy child, they were also unable to predict what the future might hold for the child. Experts understood that parents want more definitive information about the future of the child when faced with this uncertainty especially in regards to their independence.

*HCP1 (genetic counselor): They often have their own visions on who their child is going to be and what pattern they will follow and then very early on in that journey you’re informed that your child is going to take a different path so that can be very difficult of course.*

*HCP2 (speech-language pathologist): Everyone is expecting a healthy baby and they deal with the reality and the challenges of not having that so, I mean, we have a great team in terms of social worker and genetic counselors and families who help support other families along this journey but it’s challenging*.

*HCP4 (psychologist)*: *All parents want information and that’s why they come to our clinic because they want better information about how their child is functioning and they want to understand what to expect in their future so they want information how to understand their child’s abilities at school*, *what’s going to help them at school*, *a lot questions about you know are they going to be always in special education*? *Are will they ever grow up and get a job or live by themselves or got to college or university*? *So a lot of them want to know what the future holds for their child*, *will they ever you know be able to function independently or will they always want help from someone*.

Participants also acknowledged that parents are somehow stuck in a vicious circle due to the fact that, with age, new medical features might emerge that raise new concerns. When children are young, the focus is more on their physical well-being including cardiac and oropharyngeal or feeding problems. But, with time, parents’ attention seems to shift more towards neurodevelopmental issues, including speech/language and learning difficulties during the school years, differentiating them from their peers and continuing to the teenage years where behavioral problems, difficulties with social interactions and mental health issues can impact independence, friendships and family interactions.

*HCP4 (psychologist): And it takes time to work through it and to start to adjust to it but some people say well you know, I never really adjust because there’s always something new coming and there’s always something I’m concerned about, and I feel sad for my child for what’s he’s going through and just it’s made life difficult in a lot of ways for us.*

*HCP8 (genetic counselor)*: *So in terms of the present*, *I would say that’s dependent on the age and stage of the child*, *and that varies tremendously*, *so in the newborn period*, *it is just getting them healthy again*, *it’s extremely medically based those concerns in the newborn period*. *As they become toddlers and they’re starting to require language and all of their developmental skills (…) But then*, *as they enter school the concerns are often around either social immaturity and not really being able to meet those social queues with their peers*, *their concerns are about not necessarily being poorly behaved but missing important foundational academic teaching*, *it’s just going over their head and they’re not getting it*, *you know having difficulty with speech language and communication is huge and universal*. *And then as the kids get older*, *a lot of concerns around behavior*, *whether it’s poor self-regulation or more behaviors around being introverted and not wanting to socialize with other kids*, *a lot of parents worry that as their kids get older that they tend not to want to have friends in general*, *there’s definitely some kids with 22q11DS who are social butterflies but in general they don’t seem to want or seek friendships and that’s very concerning to parents as well*.

Furthermore, many HCPs acknowledged families’ fear about the increased risk of mental health issues of their child and expressed the importance of providing parents with information about their child’s neuropsychiatric status.

*HCP9 (psychiatrist): Once you have a developmental layer, I say often the profile and labels and symptoms are at risk for ID, ADHD traits, restricted behaviors and rigidity, and autism, you need a continuous construct description for diagnostic assessment to help them understanding, many families are also understandably anxious about psychosis and schizophrenia risk.*

*HCP10 (ENT surgeon)*: *I think if you have a young child with the diagnosis and you know all the possible manifestations of the syndrome and you do not know what is going to happen with your child*, *it is of course I think it is terrifying to know*, *mainly the mental well-being and all the things that can happen*, *yeah I think it is a big worry for families*.

One participant however mentioned that not all HCPs are aware of the impact of children’s constant changing health conditions on families. This is especially the case for those who have less familiarity with 22q11DS. And even those who do acknowledge families’ existential uncertainty, they might not have enough time or they might avoid the difficulty of addressing these complexities and so prefer to not tackle those challenging conversations.

*HCP3 (psychologist): If you have a child with all these things or a young adult, it’s like in periods of your life you are struggling with a lot of things, then there is a period that you think ok now everything is fine and then suddenly something happens again, so those parents, those families go through all these cycles and I really think recognizing that and acknowledging that, that is the first step. And a lot of professionals are not always aware of this from the beginning and parents feel it (…) But a lot of professionals are very afraid of this, to bring this to the table and also sometimes, they say I do not have the time to have all these conversations with the parents.*

#### 2.2. Reacting to existential uncertainty

Several professionals expressed humility in their interviews. Some felt humbled by their patients’ lived experiences and noted the importance of incorporating a family’s perspectives into any decision-making while others felt humbled by the resilience of parents facing the challenges of 22q11DS, doubting their own ability to cope in a similar situation. Many expressed frustration and guilt when feeling unable to guide parents effectively through the uncertainties of the condition, fearing they might overwhelm them with complex information or not provide enough support.

*2*.*2*.*1*. *Humbling experience*. Numerous professionals displayed a sense of modesty throughout the interviews. In one interview, the participant described themselves as a “sidebar character” supporting the main characters in a play and providing them the right recommendations to prepare them for upcoming difficulties. Another expert stated how they learned from their patients rather than the other way around, because for them 22q11DS is a lived experience and therefore they know better what helps them and what could be hurtful or paralyzing. They described an event that reminded them to always consider the families’ perspective before coming to a conclusion on what is best for their patients.

*HCP17 (occupational therapist): I think we’re like sidebar characters in a play, we’re not the main event, like you’re there, like the helper dog but not really the main show, because the main people who actually implement the plans, are the person and the parents, and you can make recommendations, you can help them get connected to things but at the end of the day, who goes home and deals with the consequences are those two people who live that life, you don’t live that life.*

*HCP1 (genetic counselor)*: *I was sharing my perspective and how important it is and that I haven’t had an adult you know who’s been anything but positive about receiving the diagnosis*, *and then a young adult in the audience stood up and she herself acknowledged that when she found out her diagnosis (…) she fell into a deep depression and it was very hard on her*. *So that was a real reminder for me that my perspective or my experience is not necessarily universally right*? *and to remind myself to be careful when presenting information*.

Others spoke about the humbleness born from witnessing the resilience of parents and did not imagine how themselves, if put in the same situation, would be able to cope. The same participant felt relieved for being seen by families as the “savior” who fixes things and for not having to deal with complex situations of existential uncertainty.

*HCP5 (ENT surgeon): There are some families who are incredibly resilient and you know you wonder how they can manage through these very very challenging circumstances (…) I can tell you, if I knew that I have a child with 22q11DS that was my own child, I would have challenges dealing with it, just from a mental point of view, just because I know a lot about the condition (…) But for the most part I think I’m lucky because I don’t have to tell them about all of the things, all of the potential genetic issues the learning issues the psychiatric issues, you know I just focus on a very small area so I don’t necessarily have to have those challenging conversations with people. I’m usually seen more as the savior because I’m a surgeon and I can come in and I can fix it.*

*2*.*2*.*2*. *Feeling of powerlessness*. Many professionals expressed their frustration when feeling unable to guide parents through the uncertainty of 22q11DS. They expressed concerns about overwhelming families with unsure and worrying information.

This was brought up in particular when speaking about the risk of onset of psychosis.

*HCP13 (clinical immunologist): The thing is this, I do not know how, let’s say you have a 22q11DS child with heart problems, they recover, they go through all that and let’s say they’re reasonably adapted but the real challenge is that we know there is a high incidence of psychosis and schizophrenia, I do not know, I would find it difficult to tell people who go through all that you might want to look out for that.*

A few participants were self-critical if they had the impression that they were not supporting or guiding the parents as they should. Often, they felt guilty for not being able to simplify an overly complicated situation and blamed themselves if their patients are dissatisfied with their care.

*HCP15 (psychiatrist): (…) I think families they do what they can, if they do other than what you have proposed, you have to question yourself: what has happened? What did they not understand? Didn’t I not explain it well? What didn’t I take into account?*

### 3. Acting on uncertainty

HCPs strived to provide tailored support to each child and family while fostering open communication, offering resources to help parents navigate their journey with resilience and on integrating the multidisciplinary teams in the caregiving process.

#### 3.1. Guiding families

Almost all participants mentioned their role of helpers through the challenging course of the condition. Whether by guiding, supporting, listening, their main goal was to reassure families and provide them with the necessary information to move forward. Constant monitoring of the clinical psychological situation was considered essential to avoid further complications.

*HCP11 (pediatrician): I think often at the beginning for families to have to come to terms with what they’re dealing with and the support they need, and you know try to help them deal with the insecurity or the uncertainty that is at the core of a lot of the way that we felt we tried to deal with 22q11DS, because you know so many things can happen on the way and in a different way from many other conditions.*

*HCP18 (psychologist)*: *The psychiatric aspect can change very quickly and there could be really like the psychiatric decompensation that can happen quickly*, *and that you (the healthcare professional) need to react very fast in order to keep the situation under control*. *And I think that’s something very challenging and that’s always ups and downs because you think that at some point that the situation is under control and that things are going well and then suddenly there is something going on and you need to react so it is always a bit like that*. *And in French we say we have to watch things like the milk on the oven*, *I do not know what would be the translation in English because it can swing out of the pan very quickly so it’s a bit like that*.

HCPs tried to present the information in a systematic way so as not to overwhelm parents, by focusing on the pressing and sometimes urgent clinical problems of the present and delaying the psychiatric concerns to the future.

*HCP1 (genetic counselor): Usually, if the child had a diagnosis in infancy it’s because there are some medical issues or problems to attend so I talk about these medical issues, but I also talk about the developmental trajectory in a general way like the risk for delays and learning difficulties (…) I do not talk too much about mental health necessarily in that first session, but I have the benefit of knowing I’m going to be seeing them again right? so I think there is only so much that people can digest and in one session and I’m already overwhelming them with information.*

*HCP11 (pediatrician)*: *The clinician in a sense is the channel for information and you try to establish a relationship before you hit them with everything*, *you don’t hit them with everything to begin with*, *we try to be gradual*.

Some interviewees believed it to be empowering for families to focus on the present, addressing current concerns, utilizing existing knowledge and interventions rather than worrying about the possibility of psychiatric decompensations in the future. The latter was perceived as a topic to discuss with parents of preadolescents and young teenagers rather than small children.

*HCP18 (psychologist): I think for us as professionals it’s very important to acknowledge this fear but also to put it in context that it’s a question that does not really concern them for now. And it’s impossible that when we see a child who’s 4 years to say how it’s going to be as an adult and that’s ok, we can worry about that but it’s probably not going to solve anything for now. But it is also a bit trying to delay those questions, because those are very important questions but probably not for a while.*

HCPs also mentioned they encouraged families to connect with other families dealing with similar challenges. They recognized the limitations of their professional expertise compared to other parents’ personal experience on the uncertainty and challenges caused by the condition. They described finding the support of a community as valuable and an important opportunity for the families to learn from each other.

*HCP7 (nurse coordinator): What I say to my families, we as healthcare professionals, we can give you the information we’ve got a lot of information but we don’t live it, you know, we don’t live this every day at home. So families do have definitely experience and knowledge and just general information on how to live that whatever disease it is, and lots of words of wisdom, and so those connections are really crucial for families.*

*HCP5 (ENT surgeon)*: *I don’t think it’s a major issue*. *I think one of the major things that I’ve learnt*, *when I see a child who has a diagnosis whether it’s 22q11 deletion or something else*, *we have a huge advantage that here I put the family in touch with other families who have children similarly affected*. *And I think listening to other families’ experience has been really really helpful because I could talk to the families about what it’s like to go through the surgery and to go through the recovery phase from a surgeon’s point of view but I don’t know what it’s like from a family’s point of view*.

#### 3.2. Role of multidisciplinary teams

Many HCPs emphasized the role of multidisciplinary and 22q11DS teams in sharing the uncertainty of the condition with the families. They viewed it valuable to work within a group of specialists to help patients and their families understand the information.

*HCP6 (speech-language pathologist): It’s overwhelming I think for most of the families especially when it’s a new diagnosis but I find that that changes a lot once they’ve met with the 22q11 team. So it’s really that initial period between the blood work being requisitioned by our surgeon, the diagnosis being communicated, us having further discussions with the family until they’ve met with the 22q11 team. Once they’ve met with them, I find that for the majority of the families that is a really good experience for them in terms of them having a better understanding of the bigger picture.*

*HCP5 (ENT surgeon)*: *And then there are other challenges*, *the psychosocial challenges because children who are 8*, *9 or 10 years old*, *they don’t want surgery right*? *why would they want to go and have an operation that’s going to make them feel sore and pain in their throat and so again we are very fortunate where I work in (name of clinic) because we have very good child life therapists and we have a very good preoperative team that will educate the families and children about what life is like in surgery and what’s like afterwards so that really helps*.

They also appreciated discussions with colleagues on the best healthcare management plan to try and address the challenges behind some of the clinical features. For example, on the necessity of nontraditional drug usage in the case of certain psychiatric manifestations in pediatric patients, one expert emphasized on the importance of dynamic constant collaboration between the many teams caring for a child with 22q11DS to provide families with guidance on what to observe in their child’s behavior and how to address it to the caring team and on how to follow up on the effects and side effects of pharmacological therapies. Another expert mentioned the importance of multidisciplinary collaborative team care in order to guide parents to the right professionals to help them.

*HCP9 (psychiatrist): (…) So it falls on the multidisciplinary teams to anticipate guidance a little bit around that so an example would be when treating ADHD this nuance about stimulants and the child with a risk for psychosis, based on the evidence we have we don’t see it increases the risk but the evidence is observational, and here’s what you would watch for and let’s check closely and see pros and cons, and think about more nuanced risk benefit discussion when prescribing psychiatric medications of some caliber to these kids (…) Part of our standard feedback had included counseling about anticipatory challenges and depending on the family, we would be more or less specific about it, but in general we would always bring that up, vulnerability for psychiatric conditions in adolescence and what to watch for, and who to connect with and so mostly the counseling had to do with transitioning and stressors.*

*HCP6 (speech-language pathologist)*: *I think once they have an understanding of 22q11DS*, *and after speaking with us they get a better understanding of how the speech stuff will be managed but then there’s also the concerns on how language will impact academics and learning so that’s another concern that sometimes gets expressed by parents (…) So I think that’s why it’s very important to have a team approach to care for these kids because it allows us to be able to guide patients and families to the right professionals who can support them in their particular area of concern particularly when it gets beyond our scope of practice*, *it’s really helpful knowing that we have that team to be able to rely on*.

## Discussion

This present study offers a valuable contribution to the limited literature on the lived experiences of HCPs caring for children with rare diseases. Our study results show that uncertainty is a common red thread that runs through the experiences of the participants. To some extent, this is not surprising. There is a great deal of uncertainty surrounding 22q11DS and rare diseases in general, due to the limited understanding and limited number of individuals with these conditions, compounded by their diverse and often unpredictable clinical presentations, diagnosis, prognosis, and treatment [16].

We were able to differentiate between epistemic and existential uncertainty. Although many taxonomies for uncertainty in healthcare have been proposed [17–19], little has been written about this distinction, especially in rare diseases. Dwan et al. proposed a conceptual design for existential uncertainty [20] and applied it to the field of oncology [21], highlighting how a serious diagnosis can challenge patients’ identity and sense of meaning by disrupting their ability to manage the ineradicable uncertainty of their being-in-the-world. Whether our participants were aware of the concept of existential uncertainty, that we do not know.

Epistemic uncertainty involves a lack of reliability, credibility, or adequacy of information about probability on the diagnosis, prognosis, causal explanations, and treatment recommendations [18, 22]. As shown in the literature, epistemic uncertainty in RDs regarding clinical presentation and treatment is a frequently discussed topic among HCPs and patients, due to the paucity of research in this area and the scarce availability and accessibility of orphan drugs [10, 23–25]. The clinical variability between individuals and the unpredictability of the progression of 22q11DS throughout an individual’s lifespan was clearly acknowledged by the experts that we interviewed and, like in the literature [4] identified as a persistent challenge.

Moreover, mental health has been a significant focus in 22q11DS because individuals affected by the condition have high rates of psychiatric disorders [26]. Although our experts underlined the importance of detecting early signs of psychiatric issues to act quickly, they found it difficult to address this topic and preferred delaying the discussion until later, so as not to overwhelm families who were already inundated with a large volume of new information to process. Our findings were not consistent with those reported in the existing literature. In fact, research with parents of children with 22q11DS shows that parents value awareness of possible mental health problems [27] and information about protective factors and resources on coping. They would also like to be aware of their child’s risk of developing a psychiatric disorder [28, 29], and of the available mental health services. This information was completely missing or was not provided by HCPs, but was obtained from conferences and the internet [27, 28]. Research in fact shows that patients and caregivers of rare childhood genetic diseases appreciate information at any time, even if it is uncertain, but they prefer to receive it from their children’s treating physician and in an accessible way [30].

To better respond to patients’ needs and intervene adequately, it is a key duty for HCPs to better understand what exactly is causing uncertainty for patients. For example, providing more information to patients is sometimes ineffective as individuals may still struggle with uncertainty due to the inherent unpredictability and complexity of medical situations. Likewise, mass information, when non-selective, can sometimes be harmful and overwhelming. It can create false expectations and exacerbate feelings of uncertainty rather than alleviate them [10]. Therefore, one can argue that our participants’ approach to postpone some information to a later time could be an appropriate strategy to help families cope with uncertainty. Delaying certain information may prevent HCPs from overwhelming patients and families, and allow them to process information in manageable amounts and at appropriate times. Nevertheless, HCPs need to walk a fine line between respecting the right to know of and protecting their patients, and may need to designate a time and a person (i.e. psychiatric genetic counselors) to discuss mental health with families as early intervention is key to prevent psychiatric complexities [27, 28].

In addition to the well-known epistemic uncertainty, the respondents in our study particularly highlighted the challenges of witnessing the existential uncertainty of families. In the existing literature, this topic is typically only discussed from the perspective of patients and informal caregivers [31, 32], omitting the viewpoint of HCPs.

With regard to existential uncertainty, many of the interviewed experts felt frustrated. This is in line with other research that shows that HCPs caring for patients with rare diseases feel discouraged when they cannot live up to patient’s expectations, especially when the result of the treatment cannot be guaranteed [10]. Many of our participants acknowledged the families’ fear of their children not reaching developmental milestones. This concern has been reported in other studies with parents and patients as well [33, 34].

A major category of uncertainty management strategies is relationship-focused, that is, directed not at ignorance, uncertainty, or psychological responses to uncertainty but at social relationships between physicians, other health professionals, and patients. These differ from ignorance-focused information-seeking strategies in relating with other persons not as a means of curing uncertainty (eliminating) but of palliating (accepting and ameliorating) its aversive psychological effects [22]. Similarly, in his paper, Berger emphasized the communicative strategies to explicitly recognize and incorporate uncertainty into the shared-decision making process [35]. Balancing honesty and hope by HCPs with a willingness to readdress any changing information can help patients navigate uncertainty [35]. How to achieve this is an ethical question that remains unanswered by HCPs who argued how much and when to deliver specific information to their patients.

Many of our participants reacted with humbleness to existential uncertainty because they were conscious about the limitations of their expertise and the importance of the lived experience of patients and families. In their paper on uncertainty from HCPs perspective in the settings of acute care, Han et al. described humility as “a second key capacity in the evolution of the physicians’ uncertainty tolerance” [22]. This is comparable to what we found: our experts seemed to show a better tolerance of uncertainty and a greater openness to communicate with colleagues within MDTs [22]. This raises an important question on whether all HCPs are actually being prepared to cope with uncertainty in clinical settings and how to prepare them well to support their patients. In their narrative review, Scott et al. presented various coping strategies for clinicians suggesting there are ways to develop skills in managing uncertainty [36].

In the realm of rare diseases, teamwork and multidisciplinary collaboration are paramount from the perspectives of patients and HCPs [10, 37, 38]. According to our participants, teamwork is essential for navigating the complexities inherent in 22q11DS. By pooling together their expertise and resources, MDTs can overcome diagnostic challenges, develop tailored management plans, and provide coordinated care [10]. By working collaboratively, HCPs can ultimately improve their patients’ quality of life as they consider not only their medical needs but also their psycho-social well-being [37]. Nevertheless, in one study healthcare providers admitted that integrating all specialties and experts in one team is not always easy [25]. This challenge was not mentioned by our participants, probably because they are familiar with working within a MDT.

Furthermore, research shows that individuals and parents of children with rare diseases often emphasize the role of peer support in helping them cope with diagnostic uncertainty and sharing information about current and future needs [39, 40]. Studies also show that both online and in-person peer support are considered effective resources for parents to navigate the healthcare system during their child’s illness journey, from seeking, to receiving, to adjusting to the rare disease diagnosis [41]. Research shows that although pediatricians often talk about the importance of peer support for parents and children with rare diseases, they are not always sure where to find them [42]. Hesitancy about peer matching can also be found in other healthcare contexts and is often driven by privacy and confidentiality issues and time management [43]. However, often it is also a sign of HCP’s desire for medical control as they worry that peer contact may incite families to look for second opinions and thus ultimately affect their trust in the healthcare team [44]. Our participants instead not only insisted on the essential role of peer support for families but were also proactive in suggesting which groups to join. This finding shows our experts’ uncertainty tolerance and their ability to manage it.

Finally, education was not addressed in our results, probably because of the high level of expertise of the professionals we interviewed. However, in the literature, HCPs express a lack of preparedness to deal with rare disease as when it is not their primary specialty [45] and a lack of preparedness to deal with medical uncertainty all together which could be further tackled by medical education and improved curricula building [45–47]. Caregivers of individuals with rare diseases and patients themselves see a big impact of medical knowledge and expertise on their overall well-being and quality of life [48–50].

### 1. Limitations

Due to the rarity of this condition, our recruitment was constrained in terms of geographical distribution of HCPs and their specialties. Moreover, most of our participants are highly experienced HCPs who are affiliated with specialized clinics focusing on genetic diseases, including 22q11DS. Although these factors pose challenges in generalizing the findings to all healthcare providers who support families with a child affected by 22q11DS, reporting the perspective of highly qualified experts remains essential to address the challenges encountered in 22q11DS.

## Conclusion

Our study highlights valuable insights on the lived experience of HCPs in witnessing and managing uncertainty in 22q11DS, a rare disease. The results of our study provide additional, empirically supported input on how experts in the field cope with epistemic and existential uncertainty, including the uncertain mental health evolution. Healthcare providers caring for families with 22q11DS reacted to uncertainty with a sense of humbleness. They promoted peer support and acknowledged the limit of their medical expertise and the need for families to share their own perspective regarding coping and management strategies. Our participants also emphasized the critical role of MDTs and showed an openness in communicating between colleagues. These strategies represent important ways of coping with uncertainty and can be further supported by medical education and trainings. Sharing the perspective of HCPs caring for families with rare diseases on how to navigate, react and act on uncertainty is a crucial topic that would benefit from further exploration in the literature.

## References

[1] Shprintzen RJ. Velo-cardio-facial syndrome: 30 Years of study. Developmental Disabilities Research Reviews. 2008;14(1):3–10. doi: 10.1002/ddrr.2 18636631 PMC2805186

[2] Blagojevic C, Heung T, Theriault M, Tomita-Mitchell A, Chakraborty P, Kernohan K, et al. Estimate of the contemporary live-birth prevalence of recurrent 22q11.2 deletions: a cross-sectional analysis from population-based newborn screening. CMAJ Open. 2021;9(3):E802–E9. doi: 10.9778/cmajo.20200294 34404688 PMC8373039

[3] Cortés-Martín J, Peñuela NL, Sánchez-García JC, Montiel-Troya M, Díaz-Rodríguez L, Rodríguez-Blanque R. Deletion Syndrome 22q11.2: A Systematic Review. Children (Basel). 2022;9(8). doi: 10.3390/children9081168 36010058 PMC9406687

[4] McDonald-McGinn DM, Sullivan KE, Marino B, Philip N, Swillen A, Vorstman JAS, et al. 22q11.2 deletion syndrome. Nature Reviews Disease Primers. 2015;1(1):15071. 10.1038/nrdp.2015.71 PMC4900471 27189754

[5] Wahrmann S, Kainulainen L, Kytö V, Lempainen J. Childhood manifestations of 22q11.2 deletion syndrome: A Finnish nationwide register-based cohort study. Acta Paediatr. 2023. doi: 10.1111/apa.16737 36867048

[6] Óskarsdóttir S, Boot E, Crowley TB, Loo JCY, Arganbright JM, Armando M, et al. Updated clinical practice recommendations for managing children with 22q11.2 deletion syndrome. Genet Med. 2023;25(3):100338. doi: 10.1016/j.gim.2022.11.006 36729053

[7] Fung WLA, Butcher NJ, Costain G, Andrade DM, Boot E, Chow EWC, et al. Practical guidelines for managing adults with 22q11.2 deletion syndrome. Genetics in Medicine. 2015;17(8):599–609. doi: 10.1038/gim.2014.175 25569435 PMC4526275

[8] Swillen A, McDonald-McGinn D. Developmental trajectories in 22q11.2 deletion. Am J Med Genet C Semin Med Genet. 2015;169(2):172–81. doi: 10.1002/ajmg.c.31435 25989227 PMC5061035

[9] Vo OK, McNeill A, Vogt KS. The psychosocial impact of 22q11 deletion syndrome on patients and families: A systematic review. Am J Med Genet A. 2018;176(10):2215–25. doi: 10.1002/ajmg.a.38673 29575505 PMC6221171

[10] Garrino L, Picco E, Finiguerra I, Rossi D, Simone P, Roccatello D. Living with and treating rare diseases: experiences of patients and professional health care providers. Qualitative Health Research. 2015;25(5):636–51. doi: 10.1177/1049732315570116 25667160

[11] Kuo DZ, Houtrow AJ, Arango P, Kuhlthau KA, Simmons JM, Neff JM. Family-centered care: current applications and future directions in pediatric health care. Matern Child Health J. 2012;16(2):297–305. doi: 10.1007/s10995-011-0751-7 21318293 PMC3262132

[12] Bassett AS, McDonald-McGinn DM, Devriendt K, Digilio MC, Goldenberg P, Habel A, et al. Practical guidelines for managing patients with 22q11.2 deletion syndrome. J Pediatr. 2011;159(2):332–9.e1. doi: 10.1016/j.jpeds.2011.02.039 21570089 PMC3197829

[13] Baylis A, Hickey S, Smith A, Kirschner R. A Holistic Approach to Care for Children with 22q11.2 Deletion syndrome: Reflecting on 10 Years of Family-Centered Team Care. Cleft Palate-Craniofacial Journal. 2022;59(4 SUPPL):18.

[14] Braun V, Clarke V. Using thematic analysis in psychology. Qualitative Research in Psychology. 2006;3(2):77–101.

[15] Braun V, Clarke V. Reflecting on reflexive thematic analysis. Qualitative Research in Sport, Exercise and Health. 2019;11(4):589–97.

[16] Bailey KM, Sahota N, To U, Hedera P. “Because it is a rare disease…it needs to be brought to attention that there are things out of the norm”: a qualitative study of patient and physician experiences of Wilson disease diagnosis and management in the US. Orphanet J Rare Dis. 2023;18(1):158. 37349760 10.1186/s13023-023-02778-3 PMC10288732

[17] Babrow AS, Kasch CR, Ford LA. The many meanings of uncertainty in illness: Toward a systematic accounting. Health Communication. 1998;10(1):1–23. doi: 10.1207/s15327027hc1001_1 16370987

[18] Han PK, Klein WM, Arora NK. Varieties of uncertainty in health care: a conceptual taxonomy. Med Decis Making. 2011;31(6):828–38. doi: 10.1177/0272989x11393976 22067431 PMC3146626

[19] Marks H, Coleman M, Michael M. Further Deliberations on Uncertainty in Risk Assessment. Human and Ecological Risk Assessment: An International Journal. 2003;9(6):1399–410.

[20] Dwan C, Willig C. Existential uncertainty in health care: A concept analysis. J Eval Clin Pract. 2021;27(3):562–70. doi: 10.1111/jep.13536 33474766

[21] Dwan C, Willig C. Existential uncertainty in the patient cancer experience: Delimiting the concept. Palliative and Supportive Care. 2023;21(2):247–53. doi: 10.1017/S1478951522000104 35166199

[22] Han PKJ, Strout TD, Gutheil C, Germann C, King B, Ofstad E, et al. How Physicians Manage Medical Uncertainty: A Qualitative Study and Conceptual Taxonomy. Med Decis Making. 2021;41(3):275–91. doi: 10.1177/0272989X21992340 33588616 PMC7985858

[23] Bryson B, Bogart K, Atwood M, Fraser K, Locke T, Pugh K, et al. Navigating the unknown: A content analysis of the unique challenges faced by adults with rare diseases. Journal of health psychology. 2021;26(5):623–35. doi: 10.1177/1359105319828150 30786780

[24] Hoffman-Andrews L. The known unknown: the challenges of genetic variants of uncertain significance in clinical practice. J Law Biosci. 2017;4(3):648–57. doi: 10.1093/jlb/lsx038 29868193 PMC5965500

[25] Reiff M, Ross K, Mulchandani S, Propert KJ, Pyeritz RE, Spinner NB, et al. Physicians’ perspectives on the uncertainties and implications of chromosomal microarray testing of children and families. Clin Genet. 2013;83(1):23–30. doi: 10.1111/cge.12004 22989118 PMC3527693

[26] Hoeffding LK, Trabjerg BB, Olsen L, Mazin W, Sparsø T, Vangkilde A, et al. Risk of Psychiatric Disorders Among Individuals With the 22q11.2 Deletion or Duplication: A Danish Nationwide, Register-Based Study. JAMA Psychiatry. 2017;74(3):282–90. doi: 10.1001/jamapsychiatry.2016.3939 28114601

[27] Carrion P, Semaka A, Batallones R, Slomp C, Morris E, Inglis A, et al. Reflections of parents of children with 22q11. 2 deletion syndrome on the experience of receiving psychiatric genetic counseling:‘Awareness to Act’. Journal of Genetic Counseling. 2022;31(1):140–52. 34224608 10.1002/jgc4.1460

[28] Alugo T, Malone H, Sheehan A, Coyne I, Lawlor A, McNicholas F. Development of a 22q11DS psycho-educational programme: exploration of the views, concerns and educational needs of parents caring for children or adolescents with 22q11DS in relation to mental health issues. Child: care, health and development. 2017;43(4):527–35. doi: 10.1111/cch.12457 28370163

[29] Blankenship K, Chieffo S, Morris E, Slomp C, Batallones R, Prijoles E, et al. Development and evaluation of an educational resource for parents of children with 22q11. 2 deletion syndrome about the psychiatric manifestations of the condition. Journal of Genetic Counseling. 2023. doi: 10.1002/jgc4.1779 37658574

[30] Pollard S, Weymann D, Dunne J, Mayanloo F, Buckell J, Buchanan J, et al. Toward the diagnosis of rare childhood genetic diseases: what do parents value most? European Journal of Human Genetics. 2021;29(10):1491–501. doi: 10.1038/s41431-021-00882-1 33903739 PMC8484431

[31] Kesselheim AS, McGraw S, Thompson L, O’Keefe K, Gagne JJ. Development and Use of New Therapeutics for Rare Diseases: Views from Patients, Caregivers, and Advocates. The Patient—Patient-Centered Outcomes Research. 2015;8(1):75–84. doi: 10.1007/s40271-014-0096-6 25362528

[32] von der Lippe C, Diesen PS, Feragen KB. Living with a rare disorder: a systematic review of the qualitative literature. Mol Genet Genomic Med. 2017;5(6):758–73. doi: 10.1002/mgg3.315 29178638 PMC5702559

[33] Ayoub S, Elger BS, De Clercq E. Undeleting the voice of people with 22q11 deletion syndrome: A scoping review. Rare. 2024;2:100033.

[34] Goodwin J, McCormack L, Campbell LE. Positive and Negative Experiences of Parenting a Pre-school Child with 22q11.2 Deletion Syndrome. Advances in Neurodevelopmental Disorders. 2017;1(2):63–72.

[35] Berger Z. Navigating the unknown: shared decision-making in the face of uncertainty. J Gen Intern Med. 2015;30(5):675–8. doi: 10.1007/s11606-014-3074-8 25536912 PMC4395589

[36] Scott IA, Doust JA, Keijzers GB, Wallis KA. Coping with uncertainty in clinical practice: a narrative review. Med J Aust. 2023;218(9):418–25. doi: 10.5694/mja2.51925 37087692

[37] Jones J, Simpson A, Meade N, Pushparajah D, Newman D, Hunter A. Rare Disease Healthcare Experience: Qualitative Survey and Workshop Findings in the Context of UK Policy Development. 2021.

[38] Walton H, Simpson A, Ramsay AIG, Hunter A, Jones J, Ng PL, et al. Development of models of care coordination for rare conditions: a qualitative study. Orphanet J Rare Dis. 2022;17(1):49. doi: 10.1186/s13023-022-02190-3 35164822 PMC8843018

[39] Grut L, Kvam MH. Facing ignorance: people with rare disorders and their experiences with public health and welfare services. Scandinavian Journal of Disability Research. 2013.

[40] Huyard C. What, if anything, is specific about having a rare disorder? Patients’ judgements on being ill and being rare. Health Expectations. 2009;12(4):361–70. doi: 10.1111/j.1369-7625.2009.00552.x 19840131 PMC5060508

[41] Baumbusch J, Mayer S, Sloan‐Yip I. Alone in a crowd? Parents of children with rare diseases’ experiences of navigating the healthcare system. Journal of Genetic Counseling. 2019;28(1):80–90. 10.1007/s10897-018-0294-9 30128673

[42] Zurynski Y, Gonzalez A, Deverell M, Phu A, Leonard H, Christodoulou J, et al. Rare disease: a national survey of paediatricians’ experiences and needs. BMJ Paediatrics open. 2017;1(1). doi: 10.1136/bmjpo-2017-000172 29637168 PMC5862166

[43] Reber N, De Clercq E. Enduring uncertainties. Medical accounts on caring for young people with variations of sex characteristics. Dialogues Health. 2022;1:100014. doi: 10.1016/j.dialog.2022.100014 38515880 PMC10953950

[44] Brodar KE, Carlisle V, Tang PY, Fisher EB. Identification and Characterization of Peer Support for Cancer Prevention and Care: A Practice Review. Journal of Cancer Education. 2022;37(3):645–54. 32892278 10.1007/s13187-020-01861-8 PMC7474572

[45] Nevalainen MK, Mantyranta T, Pitkala KH. Facing uncertainty as a medical student—a qualitative study of their reflective learning diaries and writings on specific themes during the first clinical year. Patient Educ Couns. 2010;78(2):218–23. doi: 10.1016/j.pec.2009.07.011 19767167

[46] Kim K, Lee YM. Understanding uncertainty in medicine: concepts and implications in medical education. Korean J Med Educ. 2018;30(3):181–8. doi: 10.3946/kjme.2018.92 30180505 PMC6127608

[47] Moffett J, Armitage-Chan E, Hammond J, Kelly S, Pawlikowska T. "It’s okay to not know …" a qualitative exploration of faculty approaches to working with uncertainty. BMC Med Educ. 2022;22(1):135. 35232453 10.1186/s12909-022-03180-6 PMC8887020

[48] Goodwin J, McCormack L, Campbell LE. "You don’t know until you get there": The positive and negative "lived" experience of parenting an adult child with 22q11.2 deletion syndrome. Health Psychol. 2017;36(1):45–54. doi: 10.1037/hea0000415 27657800

[49] O’Donoghue E, McAllister M, Rizzo R. The experiences of families receiving a diagnosis of 22q11. 2 deletion syndrome in Ireland. Journal of Genetic Counseling. 2023;32(3):618–34. doi: 10.1002/jgc4.1667 36575949

[50] Smits RM, Vissers E, Te Pas R, Roebbers N, Feitz WF, van Rooij IA, et al. Common needs in uncommon conditions: a qualitative study to explore the need for care in pediatric patients with rare diseases. Orphanet Journal of Rare Diseases. 2022;17(1):153. doi: 10.1186/s13023-022-02305-w 35379257 PMC8981675

